# A clustering-based approach for efficient identification of microRNA combinatorial biomarkers

**DOI:** 10.1186/s12864-017-3498-8

**Published:** 2017-03-14

**Authors:** Yang Yang, Ning Huang, Luning Hao, Wei Kong

**Affiliations:** 1 Department of Computer Science and Engineering, Shanghai Jiao Tong University, 800 Dong Chuan Road, 200240 Shanghai, China; 2 Key Laboratory of Shanghai Education Commission for Intelligent Interaction and Cognitive Engineering, Shanghai, China; 3 Department of Computer Science and Engineering, Shanghai Maritime University, 1550 Hai Gang Ave., Shanghai, China; 4 Key Laboratory of Systems Biomedicine (Ministry of Education), Shanghai Center for Systems Biomedicine, Shanghai, China

**Keywords:** MicroRNA, Biomarker, Clustering

## Abstract

**Background:**

MicroRNAs (miRNAs) have great potential serving as tumor biomarkers and therapeutic targets. As the rapid development of high-throughput experimental technology, gene expression experiments have become more and more specialized and diversified. The complex data structure has brought great challenge for the identification of biomarkers. In the meantime, current statistical and machine learning methods for detecting biomarkers have the problem of low reliability and biased criteria.

**Results:**

This study aims to select combinatorial miRNA biomarkers, which have higher sensitivity and specificity than single-gene biomarkers. In order to avoid exhaustive search and redundant information, miRNAs are firstly clustered, then the combinations of representative cluster members are assessed as potential biomarkers. Both the criteria for the partition of clusters and selection of representative members are based on Fisher linear discriminant analysis (FDA). The FDA-based criterion has been demonstrated to be superior to three other criteria in selecting representative members, and also good at refining clusters. In the comparison with eight common feature selection methods, this clustering-based method performs the best with regard to the discriminative ability of selected biomarkers.

**Conclusions:**

Our experimental results demonstrate that the clustering-based method can identify microRNA combinatorial biomarkers with high accuracy and efficiency. Our method and data are available to the public upon request.

## Background

MicroRNAs (miRNAs) play important regulatory roles in many fundamental biological processes for disease development and progression. Especially, tremendous researches have demonstrated that miRNAs can serve as oncogene or tumor suppressor in various cancer types [1, 2]. During the last decade, benefitting from the development of miRNA microarray and small RNA-Seq techniques, miRNA expression data has been widely used in the comparison of diseased samples with control samples, or different subtypes of diseased samples. The miRNAs with most discriminant capacity, regarded as biomarkers, have assisted in diagnosis, prognosis prediction and therapeutic assessment of cancers [3, 4], and sometimes they are even more accurate than coding-gene markers [5, 6].

In order to search biomarkers, the analysis of differential gene expression is performed and genes are ranked according to certain criteria. The evaluation on the quality of biomarkers is mainly based on statistical or machine learning approaches, whose corresponding measurements are statistical significance and classification accuracy, respectively.

Till now, a variety of statistical methods have been applied into the gene expression analysis. Fold change has been used as an initial metric for measuring the significance of change in expression levels between two groups of samples [7], and t-test [8] is a widely-used statistical method to select differentially expressed genes. Besides, researchers have developed many alternatives of t-test, such as ANOVA [9], Wilcoxon test [10], SAM [7], RVM [11], LIMMA [12], VarMixt [13] and SMVar [14]. Most of the present criteria are for univariate analysis. As the rapid development of high-throughput experimental technology, gene expression experiments have become more and more specialized and diversified. Especially, tissue-specific and condition-specific researches have largely been emerged. The single-gene biomarkers are often unreliable or have insufficient ability to distinguish subtypes or different conditions for complex diseases.

In order to increase the sensitivity and specificity of biomarkers, in many studies, the top ranked genes according to some selection metric were put together and used as composite biomarkers. This method is not guaranteed to find optimal biomarkers, since there may be redundant information among the genes because of correlation. And, many genes individually do not show good discriminative ability between different groups, while perform well together with other genes. Therefore, multivariate analysis is necessary to examine the performance of multiple genes as a whole. A common method for multivariate statistical analysis is Hotelling’s t-square test [15]. Note that in gene expression analysis, the number of samples is often very limited. As the dimensionality increases, the statistical inference often fails to provide reliable results.

Feature selection is a major branch of methods for screening biomarkers [16]. From a machine learning point of view, biomarkers correspond to the features with most discerning power. A multivariate feature selection method scores feature subsets and rank them, usually by their classification accuracy. For example, in order to select gene combinations, Cui et al. [17] and Xu et al. [18] used support vector machines to separate cancer and normal tissues, and assessed classification accuracy for all the *k*-gene combinations, for *k*≤4 and *k*≤8, respectively. These multivariate analysis methods can avoid feature redundancy but may run into exponential complexity due to the huge search space. Another issue is about the interpretation of computational results. Too complex classifier (often regarded as a black-box) and too many variables/features in the composite biomarkers could be useless, because the results are extremely difficult for biological explanation and validation.

MiRNA expression analysis usually follow the same procedure and approaches as mRNA expression analysis, such as hypothesis test [19], clustering [20] and classification [21] based on machine learning models. Meanwhile, the above mentioned problems also exist in miRNA data. Besides, due to the low intensity on expression level and small difference between miRNA sequences, the selection of miRNA biomarkers becomes more challenging. In this study, instead of screening single miRNAs or large miRNA sets, we aim to find the combinatorial biomarkers, i.e., *k*-miRNA combinations, where *k* is a small number. To avoid exponential number of combinations, we propose a clustering-based method to reduce the number of candidate combinations and conduct a highly efficient search. The basic idea is to assess only the combinations consisting of representative members from clusters that are generated based on expression level similarity, rather than all combinations. In order to further reduce the search space, a proper criterion is needed to rank the miRNAs in the clusters, and only the most promising ones can be selected as the representatives of their clusters to form the candidate biomarkers.

Clustering approaches have been extensively used to find co-expressed genes. Genes in the same clusters are usually functionally related. There have been some studies that adopted clustering-based methods for feature selection. For example, Jaeger et al. proposed to use a fuzzy C-means clustering method to pre-filter genes before ranking genes individually [22]. That is, only one representative gene is selected from each cluster and involved in the ranking procedure. A similar approach was proposed by Hanczar et al. [23], who used *k*-means clustering to select ‘prototype genes’. In both of these two methods, the number of clusters needs to be pre-defined. Actually, it is an important issue to find the proper number of clusters. In order to address this issue, Wang et al. developed a novel hybrid approach [24]. They applied hierarchical clustering on these genes to generate a dendrogram, then the optimal number of clusters was determined by a leave-one-out cross-validation (LOOCV) strategy by trying all of the different clusterings by breaking up the dendrogram.

In all of these methods, there is no defined criterion on how to determine the number of clusters or the proper size of clusters, though Wang et al. conducted an empirical analysis of LOOCV [24]. Moreover, these methods typically used genes which are the closest to centers of their clusters as the representative genes, while whether the center gene is the best choice is questionable. In another similar research proposed by Sahu et al. [25], *k*-means clustering was adopted, while signal-to-noise ratio (SNR score) was used to rank genes in every clusters.

Our approach has two major differences from the previous approaches: i) there is a new criterion to select the most discriminant member in each cluster, ii) there is a defined criterion to determine whether a cluster should be split. And the goal of this study is slightly different from the aforementioned literatures in that we aim to develop efficient method for identifying miRNA combinatorial biomarkers, instead of large feature subsets which are hard to be interpreted in biology. We have conducted a series of experiments to compare different criteria for selecting representative genes from clusters and splitting raw clusters. We also compared the new method with some widely used feature selections methods. The experimental results demonstrate that our proposed method is very effective in screening genes in the clusters. The resulting clusters can greatly reduce the number of combinations to be assessed, and obtain high-quality combinations in the mean time. The selected miRNA combinations have not only high discriminative ability, but also enriched pathways closely related with tumorigenesis. Moreover, many frequently present miRNAs in these combinations have been validated to be associated with breast cancer development in previous literatures.

## Methods

**Fig. 1:**
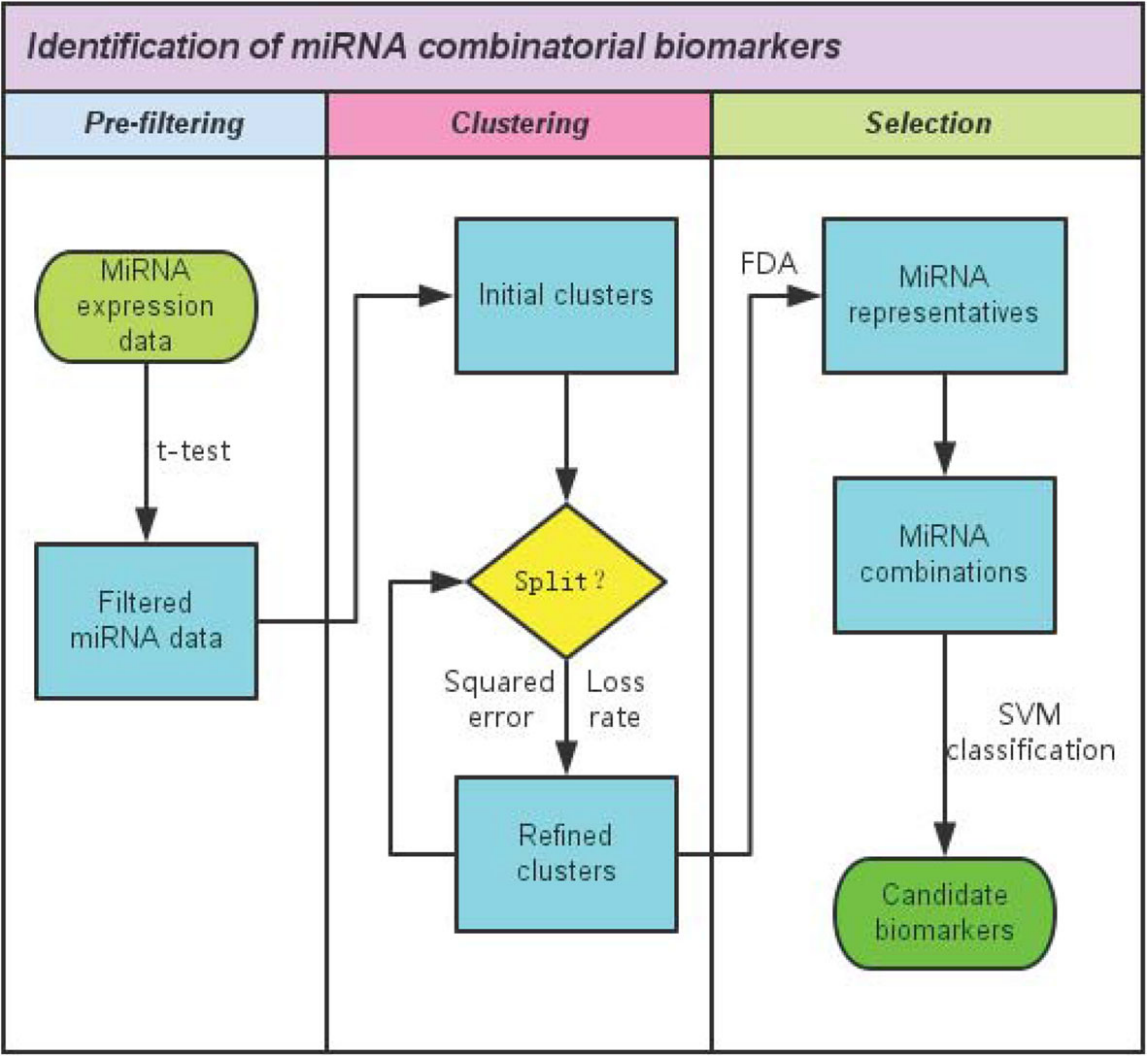
Flowchart of the feature extraction method

The proposed method consists of three major steps. The first step is a pre-screening to remove uninformative miRNAs using Welch’s t-test. The second step is a hierarchical clustering on the remaining genes. In the last step, representative miRNAs are selected from every clusters to form miRNA combinations as candidate biomarkers. Both the criteria for assessing the qualities of clustering and selecting representative miRNAs within clusters are defined through a linear discriminant method. The flowchart of the method is shown in Fig. 1.

### Fisher linear discriminant analysis

Fisher linear discriminant analysis (FDA) [26] seeks a best linear combinations of features to achieve maximum separation on the projected feature space, by optimizing the object function which is a ratio of inter-class difference to intra-class difference of data. Since FDA projects original features onto one-dimensional features, it is used not only for classification but also for dimensionality reduction. Different from principal component analysis (PCA), FDA works in a supervised manner, thus the projected features are more discriminative with respect to the classification task. The algorithm of FDA is briefly described in the below.

For a binary classification problem, suppose *X* is the training set which has *n* samples with *p* dimensions, i.e., **X**={**x**_1_,**x**_2_,**x**_3_,⋯,**x**_*n*_}, where **x**_*i*_s (1≤*i*≤*n*) are *p*-dimensional sample vectors belonging to class *c*_0_ or *c*_1_. Let *m*_0_ and *m*_1_ be the mean vectors of samples in these two classes, respectively, and **w** be the optimal projection direction. According to FDA’s object function, **w** is obtained by Eq. (1),

1 $$ \mathbf{w} \propto S_{w}^{-1}\left(\mathbf{m}_{0} - \mathbf{m}_{1}\right),  $$

where *S*_*w*_ is the sum of variance within each class, i.e.,

2 $$ {}S_{w}\,=\,\! \sum_{\mathbf{x}_{i} \in c_{0}}\left(\mathbf{x}_{i} - \mathbf{m}_{0} \right)\left(\mathbf{x}_{i} - \mathbf{m}_{0} \right)^{\mathrm{T}} + \sum_{\mathbf{x}_{i} \in c_{1}}\left(\mathbf{x}_{i} - \mathbf{m}_{1} \right)\left(\mathbf{x}_{i} - \mathbf{m}_{1} \right)^{\mathrm{T}}  $$

Given this optimal direction, all **x**_*i*_s are projected onto **w** to get the new one-dimensional sample sets **Y**={*y*_1_,*y*_2_,⋯,*y*_*n*_}, where

3 $$\begin{array}{*{20}l} {y_{i}} = {\mathbf{w}}^{T} \mathbf{x}_{i} \text{ for} \ i = 1, 2, \cdots, n \end{array} $$

As for classification, the definition of threshold (class boundary) has multiple choices. Normally, the threshold, *y*_0_, can be computed as Eq. (4),

4 $$ y_{0} = \frac{n_{0}{m_{0}} + n_{1}{m_{1}}}{n_{0} + n_{1}},  $$

where *m*_0_ and *m*_1_ are means for the two classes in the projected data space, i.e.,

5 $$\begin{array}{*{20}l} m_{0}=\mathbf{w}^{T}\mathbf{m}_{0} \end{array} $$

6 $$\begin{array}{*{20}l} m_{1}=\mathbf{w}^{T}\mathbf{m}_{1} \end{array} $$

In the test phase, a test sample **x** is firstly projected onto **w**, then the resulted value *y* is compared against *y*_0_. If *y* is larger than or equal to *y*_0_, it will be assigned to Class *c*_0_. Otherwise, it is regarded as belonging to class *c*_1_.

### The criteria for selecting representative members

In order to avoid feature redundancy, a representative member is selected from each cluster of miRNAs. Although many methods directly choose the mean or center member, it is necessary to define some criterion to rank the members by their contribution or potential in the separation of groups of samples. As described in “Fisher linear discriminant analysis” section, FDA aims to find the projection direction, **w**, with maximum discriminative capacity. And, **w** can be regarded as a vector of weights, indicating the importance of features. Intuitively, those features with the largest weights are the most informative for classification. In other words, the magnitude of each component of **w** implies the relevance of the corresponding miRNA to classification.

Let *I* be the index set of all miRNAs, i.e. *I*={1,2,…,*p*}, and *I*_*c*_ be the index set of the miRNAs in the cluster *c*. The index of the representative member of *c*, *i*_*c*_, satisfies Eq. (7):

7 $$ |\mathbf{w}(i_{c})| = \max_{j\in {I}_{c}}(|\mathbf{w}(j)|),  $$

where **w**(·) is a component of **w**.

### The criteria for splitting clusters

Besides selection of miRNAs, the determination of number or size of clusters also has a great impact on the performance of search algorithms. Too many clusters are more likely to find high quality combinatorial biomarkers but can result into huge computational complexity. The extreme case is the trivial clustering that each single miRNA is a cluster. On the contrary, too few clusters would miss valuable combinations since only a few representative miRNAs are considered. Thus here is a tradeoff between accuracy and efficiency. Instead of explicitly specify the number of clusters, we seek proper criteria for determining whether a given cluster needs to be split into smaller clusters.

Here, we define the criterion mainly based on the loss of information caused by projection. Intuitively, if the cluster members are diversified, it would be very hard to find a unified direction for projection, so the data samples would suffer great information loss after projection, which indicates that the cluster needs to be split. Thus, we define a measure called mean squared loss (*MSL*), to estimate average information loss in a cluster. Equation (8) formulates this measure.

Let *h* be the hyperplane that passes the mean point of the data samples and has normal direction of **w** (FDA projection direction), then *MSL* is defined as:

8 $$ MSL = \frac{\sum_{i=1}^{n} |\mathbf{P}_{h}(\mathbf{x}_{i}-\mathbf{m})|^{2}}{n},  $$

where **P**_*h*_(·) denotes the projection of a vector onto *h*, **m** is the mean vector of samples, *x*_*i*_ is the *i*th data sample. Since *h* is perpendicular to **w**, we regard the projection of the difference between **x**_*i*_ and **m** on *h* as an approximative loss caused by FDA projection.

Furthermore, considering that the samples may differ in data magnitudes, we define another criterion called mean loss rate (*MLR*) as shown in Eq. (9),

9 $$ MLR = \frac{\sum_{i=1}^{n} \frac{|\mathbf{P}_{h}(\mathbf{x_{i}}-\mathbf{m})|}{|\mathbf{x}_{i}|}}{n},  $$

where *MLR* denotes the averaged loss rate, i.e. the ratio of the loss (in the projection) to the norm of sample.

The whole pipeline is described in Algorithm ??, in which the *MLR* is used as the selection criterion.



### Evaluation criteria

The performance of different criteria are evaluated using two measures for the resulted combinations which are ranked top 10, 100 and 1000, respectively. One is average rank, denoted by *AvgRank*, and the other is the proportion of the true top combinations identified by the method, denoted by *HitRatio*.

These two measures are defined in Eqs. (10) and (11), respectively. For top *n* *k*-miRNA combinations searched by the method,

10 $$ {AvgRank}_{n} = \frac{\sum_{1\leq i \leq n} {rank}_{i}}{n},  $$

where *r* *a* *n* *k*_*i*_ is the true rank of the *i*th best combination identified among all *k*-miRNA combinations (In contrast of the rank obtained by the proposed heuristic search, we call the original rank of the miRNA combination by using the exhaustive search as “true rank”). All these ranks are determined according to classification accuracy.

11 $$ {HitRatio}_{n} = \frac{{hit}_{n}}{n},  $$

where *h* *i* *t*_*n*_ is the number of hits in the *n* best combinations searched by the method. A hit means the searched result is truly among the top-*n* combinations.

Apparently, small *AvgRank* and high *HitRatio* of the search results indicate good performance of the algorithm for identifying high-quality biomarker candidates.

In addition, to evaluate the classification performance of the selected miRNA combinations, we used three accuracy measures, namely sensitivity, specificity and total accuracy (TA).

## Results

### Data sets

In this study, we used two public miRNA data sets from NCBI GEO [27], GSE22220 [28] and GSE40525 [29], which were measured by Illumina Human v1 miRNA panel and Agilent-019118 Human miRNA microarray platform, respectively. Both of these two studies aim to explore function of microRNAs in breast tumorigenesis, and reveal potential therapeutic targets. There are a total of 120 samples collected from 64 breast cancer patients, including 56 pairs of matched tumor and adjacent peri-tumoral breast tissues, and 8 unmatched tissues in GSE 40525. And in GSE22220, there are 210 samples from 219 breast cancer patients, including 84 estrogen receptor (ER)-negative tissues, and 135 ER-positive tissues. The detailed statistics of patient characteristics are shown in Table 1.

**Table 1:** Sample statistics

Characteristics	GSE22220	GSE40525
Grading		
G1	42	3
G2	87	31
G3	65	27
Nodal status		
N0	127	29
N+	92	32
Estrogen receptor		
Positive	135	47
Negative	84	27

In order to ensure the data quality, we removed the miRNAs whose expression levels were not detected or below the threshold value in more than 30% of the samples. GSE40525 was classified into two categories according to tumor and peri-tumor status, while GSE22220 was divided into two categories according to ER status. Finally, the GSE40525 data set contains 52 pairs of tumor and peri-tumor profiles and the GSE22220 data set contains 127 samples of ER-positive and 80 of ER-negative.

### Experimental settings

As a pre-screening step, Welch’s t-test was conducted on the two data sets. MiRNAs with pvalue greater than 0.05 were filtered out, and the remaining miRNAs were clustered by a hierarchical clustering with average-link method. Next, the hierarchical tree was cut into raw clusters. In order to find natural cluster divisions in the hierarchical tree, we computed inconsistency coefficient for each link in the tree [30]. This value compares the height of a link in a hierarchical tree with the average height of links below it. Inconsistent links indicate the border of naturally divided clusters. The inconsistency coefficients range from 0 to 1.15 for both the two data sets. Thus we specified an inconsistency coefficient threshold of 1 to partition the two hierarchical trees into raw clusters, resulting in 82 and 63 clusters, respectively.

Further, FDA-based criteria were used to determine whether or not those clusters should be split into smaller clusters. After the final clusters were determined, a representative member was selected from each cluster to form miRNA combinations. The comparison on several criteria for selecting representative members within clusters and for splitting clusters are given in the following two sections.

In the final step, each combination was assessed by classification accuracy. We evaluated the classification accuracies of all *k*-combinations (*k*≤4) comprised by the selected representative miRNAs using LIBSVM [31] with default parameters via 5-fold cross validation. The *AvgRank* and *HitRatio* were calculated based on the true ranking lists obtained by exhaustive searches.

### Comparison of criteria for selecting representatives in clusters

In previous researches, the center gene, i.e. the gene closest to the cluster center, was usually selected as the representative member of a cluster [22–24]. Also, some researchers proposed specific ranking criteria, such as the signal-to-noise ratio (SNR) proposed by Sahu et al. [25]. Here, we compared the FDA measure with three other methods based on center-gene, SNR, and pvalue of t-test, respectively. All the *k*-miRNA combinations (2≤*k*≤4, i.e. pair, triple and quadruple) resulted from these selection criteria were assessed.

In order to evaluate the quality of the search results, we examined top 10, 100 and 1000 best combinations identified by these four methods and recorded their *AvgRank*s and *HitRatio*s obtained on GSE22220 and GSE40525 in Tables 2 and 3, respectively.

**Table 2:** Comparison of four selection criteria on GSE22220

Feature combination	Performance measure	Selection criteria			
		FDA	T-test	SNR	Center^a^
Pair	AvgRank _10_	7.6	8.2	96.7	112.2
	AvgRank _100_	82.9	84.4	412.3	557.5
	AvgRank _1000_	1586.5	1624.4	3248.7	3355.7
	HitRatio _10_(%)	80.0	70.0	0	0
	HitRatio _100_(%)	59.0	58.0	6.0	4.0
	HitRatio _1000_(%)	33.0	32.0	16.0	11.0
Triple	AvgRank _10_	9.9	8.6	333.1	333.3
	AvgRank _100_	93.4	94.4	2607.6	2270.7
	AvgRank _1000_	1612.2	1684.3	13833.3	14626.0
	HitRatio _10_(%)	60.0	50.0	10.0	0
	HitRatio _100_(%)	58.0	58.0	2.0	1.0
	HitRatio _1000_(%)	28.0	27.1	1.3	1.5
Quadruple	AvgRank _10_	12.8	12.8	41.7	744.9
	AvgRank _100_	115.4	108.5	408.2	4938.1
	AvgRank _1000_	1482.3	1562.6	10491.6	39605.2
	HitRatio _10_(%)	40.0	40.0	50.0	0
	HitRatio _100_(%)	50.0	45.0	24.0	0
	HitRatio _1000_(%)	26.2	24.4	8.4	0.5

^a^Center denotes the method using center gene as the representative member

**Table 3:** Comparison of four selection criteria on GSE40525

Feature combination	Performance measure	Selection criteria			
		FDA	T-test	SNR	Center
Pair	AvgRank _10_	9.1	9.1	39.1	57
	AvgRank _100_	103.6	103.0	335.6	402.6
	AvgRank _1000_	1469.8	1470.5	2085.6	2683.6
	HitRatio _10_(%)	40.0	40.0	10.0	0
	HitRatio _100_(%)	64.0	64.0	18.0	12.0
	HitRatio _1000_(%)	39.0	39.2	24.4	15.8
Triple	AvgRank _10_	26.3	26.3	262.8	360.2
	AvgRank _100_	229.4	229.4	1427.0	1737.2
	AvgRank _1000_	1573.9	1577.2	9085.5	11949.7
	HitRatio _10_(%)	20.0	20.0	0	0
	HitRatio _100_(%)	66.0	66.0	6.0	4.0
	HitRatio _1000_(%)	37.0	36.8	4.6	2.8
Quadruple	AvgRank _10_	174	174	229	191
	AvgRank _100_	273	273	2610.9	1906.5
	AvgRank _1000_	2836.0	2826.5	2926.7	2965.3
	HitRatio _10_(%)	40.0	40.0	20.0	60.0
	HitRatio _100_(%)	4.0	4.0	2.6	6.0
	HitRatio _1000_(%)	19.3	19.0	2.6	4.8

**Fig. 2:**
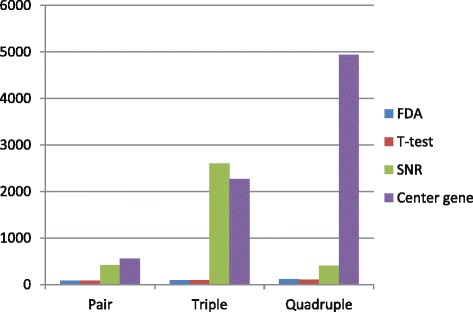
*AvgRank* of top 100 lists obtained by the four methods for GSE22220

**Fig. 3:**
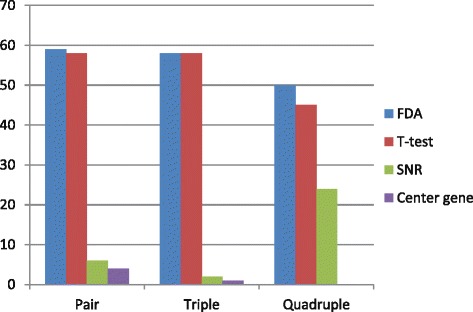
*HitRatio* of top 100 lists obtained by the four methods for GSE22220

The results show that a proper selection criterion is crucial for searching high-quality miRNA combinations. Specifically, FDA and t-test based criteria have significant advantage over other two methods, and SNR is slightly better than center-gene. For instance, on GSE22220, FDA and t-test successfully identified the best pair and triple miRNAs, and the second-best quadruple, whose accuracy is only 0.4% lower than the best one. FDA and t-test have much smaller *AvgRank*s than SNR and center-gene, no matter what the *k* is and how long the top list considered. Moreover, FDA hits 80% of the top 10 pairs. Both FDA and t-test catch majority of the top-ranked miRNA pairs and triples. As *k* increases to 4, the hit ratio decreases greatly, which is mainly due to the exponentially expanded search space. *AvgRank* and *HitRatio* values of the top 100 lists obtained by the four methods on GSE22220 data set are depicted in Figs. 2 and 3, respectively.

Generally, these methods have consistent performance on the two data sets. For GSE40525, the accuracies of combinatorial miRNAs are very high. Even a pair of miRNAs can achieve the accuracy as high as 92.3%, and the highest accuracy of quadruples is 95.2%, which suggests that the *k*-miRNA combinations (*k*≤4) are sufficient for separating the samples from two classes. The goal of GSE40525 is to discriminate tumor and peri-tumor samples, thus the differential expressed signal may be widespread. If too many combinations can achieve high accuracies, the real biomarkers may become not that notable. Thus, the results of average rank and hit ratio seem to be worse than those of GSE22220.

### Comparison of criteria for splitting clusters

**Fig. 4:**
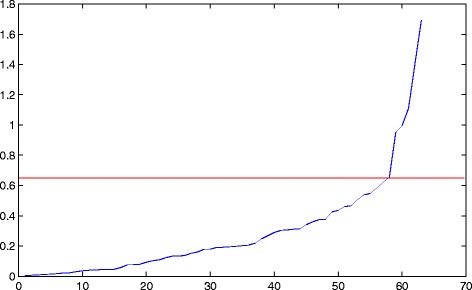
*MSL* curve of the initial clusters of GSE40525

In this study, we propose two criteria for determining whether a given cluster should be split, i.e., mean squared loss (*MSL*) and mean loss rate (*MLR*). Considering that different clusters contain different numbers of miRNAs, instead of using the original *MSL*, we divide the squared loss by *m* (number of miRNAs in the cluster), and use $MSL' = \frac {MSL}{m}$ in the analysis. The *M* *S* *L* ^′^s for all raw clusters in GSE40525 sorted in ascending order are shown in Fig. 4. It can be observed that a dramatic change occurs a little above 0.6 on the curve. Thus, we set the threshold as 0.65, where the steepest ascent locates. And we found that in GSE22220 the value is very close.

Obviously, *MLR* would grow rapidly as the number of miRNAs in the clusters increases. Here we set the threshold as $1-\frac {1}{m^{2}}$, which is a relatively loose criterion. *MLR* works as a supplement to *MSL*. In our experiment, if either of these two criteria is not satisfied (i.e., *MLR*/*MSL* is greater than its threshold), the cluster should be partitioned.

We compared the refined clustering (RC) by using these two criteria and the conventional hierarchical clustering (HC) without further splitting. The results are shown in Table 4. Considering that the results obtained after refinement are generally better than those from raw clusters because more clusters make larger search space of miRNA combinations, we did not use results of the raw clusters in the RC experiment. Instead, we tried different inconsistency coefficients for HC, which produced close number of clusters as RC did, and selected the best results, while in RC the inconsistency coefficient and thresholds of *MSL* and *MLR* are fixed as mentioned above.

**Table 4:** Comparison of clustering methods on two data sets

Feature combination	Performance measure	GSE22220		GSE40525	
		HC^b^	RC^a^	HC^b^	RC^a^
Pair	AvgRank _10_	7	8.2	8.5	9.1
	AvgRank _100_	92.2	84.4	180.9	103.0
	AvgRank _1000_	2003.1	1624.4	10696.8	1470.5
	HitRatio _10_(%)	70.0	70.0	60.0	40.0
	HitRatio _100_(%)	54.0	58.0	32.0	64.0
	HitRatio _1000_(%)	30.0	32.0	15.0	39.2
Triple	AvgRank _10_	8.2	8.6	9.2	26.3
	AvgRank _100_	95.4	94.3	68.7	229.4
	AvgRank _1000_	1776.2	1684.3	3675.2	1577.2
	HitRatio _10_(%)	60.0	60.0	20.0	20.0
	HitRatio _100_(%)	58.0	58.0	71.0	66.0
	HitRatio _1000_(%)	27.1	27.1	30.3	36.8
Quadruple	AvgRank _10_	14.2	12.8	9.0	174.0
	AvgRank _100_	112.6	108.5	257.2	273.0
	AvgRank _1000_	1639.1	1482.3	3171.3	2826.6
	HitRatio _10_(%)	30.0	40.0	78.0	40.0
	HitRatio _100_(%)	48.0	50.0	12.0	4.0
	HitRatio _1000_(%)	23.2	26.4	16.0	19.3

^a^RC: refined clustering, in which the inconsistency coefficient for raw clusters and thresholds of *MSL* and *MLR* are fixed

^b^HC: hierarchical clustering, which performs the best by trying different inconsistency coefficients

Generally, RC has a comparable or better performance to the best HC. For the top 10 list, HC shows some advantage, while for top 100 and 1000, RC performs better. These results suggest that *MSL* and *MLR* can help to improve the clustering quality, and save effort on searching good threshold to yield clusters in the hierarchical tree. Basically, both *MSL* and *MLR* measure the part of information that cannot be expressed by the projected features, i.e. information loss during the projection. Different from the absolute loss represented by *MSL*, *MLR* measures the relative loss and plays a part in screening low-quality clusters when the variances of miRNAs differ greatly.

### Comparison with existing feature selection methods

We further compared the new method with some widely used feature selection methods, including the Correlation-based Feature Selection (CFS) [32], best-first search (BFS), consistency-based selection [33], Chi-square score [34], information gain (IG) [35], Random forest (RF) filter [36], t-test [37] and the Wilcoxon rank-sum test [38].

Among these methods, CFS, BFS and consistency-based methods determine the number of selected features automatically. For other methods, we chose the subsets consisting of top 2, 3 and 4 features in the assessment. The R package FSelector [39] was adopted to implement these eight methods in the comparison experiments.

Most feature selection methods shown in Table 5 are filtering methods, except that BFS is a wrapper method, and the proposed method can be regarded as a hybrid method, which conducts filtering within clusters and then acts as a wrapper method using SVMs. The best miRNA combinations identified by the new method achieve the highest accuracies on both data sets, increasing the total accuracies by about 0.5% on GSE22220 and 1.9% on GSE40525 compared with the best accuracies obtained by other methods. This result again demonstrates the validity of clustering-based screening and the FDA criteria. Given the representative members selected from clusters, the search space is greatly reduced and the best combinations can be efficiently searched. Hence, the new method achieves a good balance between efficiency and accuracy.

**Table 5:** Comparison of feature selection methods on two data sets^a^

Methods	Feature #	GSE22220			GSE40525		
		Sensitivity	Specificity	TA	Sensitivity	Specificity	TA
CFS	29/6	0.984	0.744	0.783	0.942	0.925	0.933
BFS	4/3	0.953	0.733	0.758	0.904	0.904	0.904
*χ* ^2^	2	0.976	0.701	0.729	0.923	0.906	0.913
	3	0.913	0.753	0.763	0.923	0.923	0.923
	4	0.945	0.764	0.787	0.923	0.923	0.923
Consistency	13/5	0.953	0.771	0.797	0.942	0.925	0.933
IG	2	0.976	0.701	0.729	0.827	0.896	0.865
	3	0.913	0.753	0.763	0.942	0.925	0.933
	4	0.945	0.764	0.787	0.923	0.906	0.913
RF	2	0.976	0.701	0.729	0.923	0.906	0.913
	3	0.913	0.734	0.744	0.942	0.891	0.913
	4	0.953	0.747	0.773	0.942	0.907	0.923
t-test	2	0.913	0.753	0.763	0.923	0.906	0.913
	3	0.890	0.807	0.802	0.923	0.889	0.904
	4	0.890	0.837	0.826	0.942	0.891	0.913
Wilcon test	2	0.913	0.753	0.763	0.923	0.906	0.913
	3	0.890	0.807	0.802	0.942	0.891	0.913
	4	0.937	0.793	0.812	0.942	0.925	0.933
CluFDA^b^	2	0.969	0.750	0.783	0.923	0.923	0.923
	3	0.976	0.775	0.812	0.942	0.925	0.933
	4	0.906	0.833	**0.831**	0.962	0.943	**0.952**

^a^The numbers before and after ‘/’ denotes feature numbers of GSE22220 and GSE40525, respectively Sensitivity = *TP*/(*TP*+*FN*), Specificity = *TN*/(*FP*+*TN*) TA: total accuracy

^b^CluFDA denotes the clustering-based feature selection using FDA method for selecting representative miRNAs

### Functional analysis on the selected miRNAs

In order to perform functional enrichment on the miRNA combinatorial biomarkers, we analyzed the enriched pathways of their target genes by using mirPath [40]. For GSE40525 data set, triples of miRNAs have the best discriminant capacity, and the top 5 significant pathways for the best triple are: Fatty acid biosynthesis, PI3K-Akt signaling pathway, Prostate cancer, TGF-beta signaling pathway and p53 signaling pathway, all of which have pvalues below 5×10^−7^. For the GSE22220 data set, the enriched pathways include PI3K-Akt signaling pathway, NF-kappa B signaling pathway, focal adhesion, etc. Interestingly, PI3K-Akt signaling pathway is significantly enriched in both data sets. This pathway acts as regulator of cell proliferation, differentiation, apoptosis, and plays important roles in tumorigenesis.

In addition, we found that the top-ranked combinations usually have overlapped members. For example, all the top 10 pairs and triples of GSE40525 contains hsa-miR-139-5p. And, best quadruples often contain best pairs and triples. Therefore, we recorded the most frequent miRNAs in pairs and triples respectively and got their intersection set (Table 6). There are 8 miRNAs and 7 miRNAs for GSE22220 and GSE40525, respectively. Furthermore, these miRNAs were searched against two miRNA-disease relationship databases, namely HMDD v2.0 [41] and miR2Disease [42]. Among the 15 most frequent miRNAs, 9 miRNAs were reported in previous literatures as being involved in the development of breast cancer (Table 7). It is worth noting that 4 of the miRNAs are not covered in the top 10 list evaluated by statistical significance of the conventional t-test ranking method, but all of them have supporting literatures. Specifically, hsa-miR-365 ranks 11, hsa-miR-340 ranks 33, hsa-miR-100 ranks 34, and hsa-miR-141 ranks 56. Both miR-340 and miR-100 have been demonstrated as inhibitors of tumorigenesis with biological-experimental evidence.

**Table 6:** Most frequent miRNAs in pairs and triples^a^

GSE22220		GSE40525	
MiRNA	*P* value	MiRNA	*P* value
hsa-miR-18a*	2.09E-10	hsa-miR-139-5p	2.37E-24
**hsa-miR-146b-5p**	2.79E-10	hsa-miR-378	7.59E-20
**hsa-miR-149**	7.01E-09	hsa-miR-145	5.07E-18
**hsa-miR-224**	1.43E-08	hsa-miR-125b-2*	1.53E-14
hsa-miR-577	1.02E-07	**hsa-miR-340**	1.30E-10
hsa-miR-452*	1.51E-07	**hsa-miR-100**	1.34E-10
hsa-miR-18a	1.89E-07	**hsa-miR-141**	1.02E-08
**hsa-miR-365**	2.28E-07		

^a^MiRNAs that have evidence of association with breast cancer (from HMDD and miR2Disease) are in bold

**Table 7:** Most frequent miRNAs in pairs and triples

MiRNA name	PMID	Description
hsa-mir-18a	16754881	Copy number loss
	19684618	Higher levels of expression in ERalpha-negative tumors
	19624877	Differentially expressed between breast cancer cells and mammary epithelial cells, highly expressed in MCF-7 cells
	21755340	Expression was much higher in ERa-negative than in ERa-positive tumors.
hsa-mir-146b	16461460	Overexpressed
	19190326	miR-146: Breast cancer metastasis suppressor 1 up-regulates miR-146, which suppresses breast cancer metastasis
	18634034	miR-146: rs2910164 were associated with increased risk of breast cancer in Chinese women
	21409395	miR-146b-5p preferentially expressed in normal basal cells
	21472990	Down-regulation of BRCA1 expression by miR-146a and miR-146b-5p in triple negative sporadic breast cancers.
hsa-mir-149	18634034	miR-149: rs2292832 were associated with increased risk of breast cancer in Chinese women
hsa-mir-224	21953071	Down-regulated during lobular neoplasia progression compared to normal epithelium.
	22809510	MicroRNA-224 targets RKIP to control cell invasion and expression of metastasis genes in human breast cancer cells.
hsa-mir-452	22353773	Differentially expressed between serum samples from patients with cancer and serum samples from healthy controls
hsa-miR-365	18812439	Up-regulated greater than 2-fold in BC compared with NAT, potential target genes include members of RAS oncogenes.
hsa-mir-340	21225860	Inhibition of breast cancer cell migration and invasion through targeting of oncoprotein c-Met
	21692045	Inhibites breast cancer cell migration and invasion through targeting of oncoprotein c-Met.
hsa-mir-100	21634028	Regulates beta-tubulin isotypes in MCF7 breast cancer cells.
	22926517	Suppresses IGF2 and inhibits breast tumorigenesis by interfering with proliferation and survival signaling.
hsa-mir-141	18376396	Downregulated
	22952344	CTC (circulating tumour cells)-positive had significantly higher levels of miR-141 than CTC-negative MBC and controls.

## Discussions

In this paper, we propose to identify miRNA combinatorial biomarkers due to the important role that miRNAs play in the development of cancer and also some good properties of combinatorial biomarkers. The reasons for searching biomarkers of miRNA combinations are manifold. Firstly, single-gene biomarkers identified by uni-variate analysis are often unreliable with low specificity for discriminating complex disease properties. Thus, multi-gene biomarkers are in great need. However, the biomarkers containing too many genes, resulted from feature subset selection, are extremely difficult to be interpreted in biomedicine. For instance, if we have identified a *k*-tuple combinatorial biomarker, and we want to validate the overexpress/unexpress rule as well as inter-correlation in this biomarker, the over/under express pattern has a total of 2 ^*k*^ possibilities. Moreover, correlation coefficient can only be computed between two genes, and now there have been some studies on the conditionally independent properties in triples (3-gene combinations). But there have been no effective means to measure or validate the interconnection among multiple genes yet. Moreover, according to our results, combinations with small *k* have sufficient capability to separate groups of samples. We have also examined the accuracy of using all representative members selected from every clusters (Table 8), which are much lower than the best *k*-miRNA combinations (*k*≤4), decreasing by about 4% on GSE22220 and 9% on GSE40525. This result further demonstrates the usefulness of small combinatorial biomarkers.

**Table 8:** Accuracies of different feature subsets^a^

Feature subset	Accuracy measure	GSE22220				GSE40525			
		T-test	FDA	SNR	Center	T-test	FDA	SNR	Center
All^a^	Sensitivity	0.874	0.882	0.921	0.906	0.962	0.962	0.962	0.942
	Specificity	0.804	0.794	0.770	0.762	0.806	0.781	0.806	0.817
	TA	0.792	0.787	0.783	0.768	0.865	0.846	0.865	0.865
Pair	Sensitivity	0.969	0.969	0.929	0.984	0.923	0.923	0.885	0.846
	Specificity	0.750	0.750	0.756	0.714	0.923	0.923	0.920	0.917
	TA	0.783	0.783	0.773	0.749	0.923	0.923	0.904	0.885
Triple	Sensitivity	0.976	0.976	0.850	0.984	0.942	0.942	0.942	0.904
	Specificity	0.775	0.775	0.812	0.714	0.925	0.925	0.925	0.922
	TA	0.812	0.812	0.787	0.749	0.933	0.933	0.933	0.913
Quadruple	Sensitivity	0.906	0.906	0.906	0.890	0.942	0.942	0.887	0.923
	Specificity	0.833	0.833	0.821	0.819	0.961	0.961	0.940	0.960
	TA	**0.831**	**0.831**	0.821	0.812	**0.952**	**0.952**	0.915	0.942

^a^All: the full set of representative miRNAs selected from clusters Sensitivity = *TP*/(*TP*+*FN*), Specificity = *TN*/(*FP*+*TN*) TA: total accuracy

## Conclusions

MiRNA expression files have been widely used in the identification of biomarkers for complex diseases. Due to the low specificity of single-gene biomarker and difficulty in interpretating large gene set, this study aims to develop efficient algorithm for searching miRNA combinatorial biomarkers with high discriminability. We propose a clustering-based method to avoid brute force search, and define two types of criteria for refining clusters and selecting representative members. The former criterion aims to measure the loss during the feature projection by Fisher linear discriminant analysis, and determine whether or not to partition the given clusters. The latter criterion aims to select the most informative miRNAs in the clusters according to their contribution for classification in FDA model. We conducted experiments on two breast cancer miRNA expression profiles. The FDA-based selection method performs the best with regard to average rank of the top searched results and hit ratio on the true top list. The FDA-based cluster splitting rule has also been demonstrated to be effective in refining the clustering results. For the two data sets, *k*-miRNA combinations with *k*≤4 have sufficient capacity to discriminate the samples (83% for GSE22220 and 95% for GSE40525). This method can also be applied to the search of combinations with larger *k*, and mRNA expression data. The top-ranked miRNA combinations are worth further study on their functions as well as interactions of the miRNAs. As an additional computational analysis, the most frequent miRNAs occurring in top 10 pairs and triples have been searched again miRNA-disease database. Among the 15 most frequent miRNAs, 9 miRNAs have supporting literatures of their roles in the development of breast cancer.
